# Calculation of Direct Antiretroviral Treatment Costs and Potential Cost Savings by Using Generics in the German HIV ClinSurv Cohort

**DOI:** 10.1371/journal.pone.0023946

**Published:** 2011-09-09

**Authors:** Matthias Stoll, Christian Kollan, Frank Bergmann, Johannes Bogner, Gerd Faetkenheuer, Carlos Fritzsche, Kirsten Hoeper, Heinz-August Horst, Jan van Lunzen, Andreas Plettenberg, Stefan Reuter, Jürgen Rockstroh, Hans-Jürgen Stellbrink, Osamah Hamouda, Barbara Bartmeyer, and the ClinSurv Study Group

**Affiliations:** 1 Infectious Diseases Unit, Medical University, Hanover, Germany; 2 Robert Koch Institute, Berlin, Germany; 3 Charité University Medicine, Berlin, Germany; 4 University Hospital, Ludwig-Maximilian University, Munich/München, Germany; 5 University Hospital Cologne, Köln (Cologne), Germany; 6 University Hospital, Rostock, Germany; 7 University Schleswig Holstein, Kiel, Germany; 8 University Hospital Eppendorf, Hamburg, Germany; 9 Ifi-Institute for Interdisciplinary Medicine, Hamburg, Germany; 10 University Hospital, Düsseldorf, Germany; 11 University Hospital, Bonn, Germany; 12 ICH Studycenter, Hamburg, Germany; University of Cape Town, South Africa

## Abstract

**Background/Aim of the Study:**

The study aimed to determine the cost impacts of antiretroviral drugs by analysing a long-term follow-up of direct costs for combined antiretroviral therapy, cART,-regimens in the nationwide long-term observational multi-centre German HIV ClinSurv Cohort. The second aim was to develop potential cost saving strategies by modelling different treatment scenarios.

**Methods:**

Antiretroviral regimens (ART) from 10,190 HIV-infected patients from 11 participating ClinSurv study centres have been investigated since 1996. Biannual data cART,-initiation, cART-changes, surrogate markers, clinical events and the Centre of Disease Control- (CDC)-stage of HIV disease are reported. Treatment duration was calculated on a daily basis via the documented dates for the beginning and end of each antiretroviral drug treatment. Prices were calculated for each individual regimen based on actual office sales prices of the branded pharmaceuticals distributed by the license holder including German taxes.

**Results:**

During the 13-year follow-up period, 21,387,427 treatment days were covered. Cumulative direct costs for antiretroviral drugs of €812,877,356 were determined according to an average of €42.08 per day (€7.52 to € 217.70). Since cART is widely used in Germany, the costs for an entire regimen increased by 13.5%. Regimens are more expensive in the advanced stages of HIV disease. The potential for cost savings was calculated using non-nucleotide-reverse-transcriptase-inhibitor, NNRTI, more frequently instead of ritonavir-boosted protease inhibitor, PI/r, in first line therapy. This calculation revealed cumulative savings of 10.9% to 19.8% of daily treatment costs (50% and 90% substitution of PI/r, respectively). Substituting certain branded drugs by generic drugs showed potential cost savings of between 1.6% and 31.8%.

**Conclusions:**

Analysis of the data of this nationwide study reflects disease-specific health services research and will give insights into the cost impacts of antiretroviral therapy, and might allow a more rational allocation of resources within the German health care system.

## Introduction

The implementation of combined antiretroviral therapy, cART, as the standard of care since the middle 1990s has substantially reduced morbidity and mortality in HIV-infected individuals [1], [2], leading to decades of gain in life expectancy for these individuals, comparable to the normal age-matched population in industrialized countries [3]. Recent standard treatment guidelines recommend cART regimens in treatment-naive patients consisting of two nucleoside analogues (nRTI, and, in addition, a non-nucleoside reverse transcriptase inhibitor (NNRTI), a ritonavir-boosted protease inhibitor (PI/r) or, more recently, an integrase strand transfer inhibitor (INSTI) [4], [5], [6].

The regulation of pricing and the reimbursement of prescription medicines vary considerably between different countries and result in notable differences in the market prices of medicinal products [7]. Prices of antiretroviral drugs in Germany are high – even in comparison to other industrialized countries – due to national specifics of the pharmaceutical market. Remarkable differences in national drug prices were found during an international survey [8], but the authors did not necessarily see a correlation between purchase volume and drug prices. Therefore, the authors hypothesized that the availability of generic drugs next to branded drugs in the same market would lead to a reduction in prices.

In Germany, the yearly direct costs of HIV disease to the health care system were estimated as €24,482 per patient in 2001 [9]. But more detailed data of the direct costs for cART in the specific surroundings of the German health care system are needed to not only allow an international comparison but also an optimization of resource allocation. The following specific German conditions have to be taken into account when analysing the direct costs of cART:

- Market prices for pharmaceuticals can be calculated by the manufacturer without negotiation with the authorities or health care insurers.
- Until recently, health economic aspects were hardly considered by the German guidelines for antiretroviral treatment.
- Several health care reforms have recently been implemented in Germany to restrict the increasing expenditure. Equity and effectiveness should be enhanced by reimbursements that are calculated on the basis of lump sums for hospital stays (German disease related groups: G-DRG) and within the German risk structure compensation for health care insurance.
- In Germany physicians are obligated to the *economic efficiency principle* by the German Social Insurance Code, when they choose treatment alternatives for patients within the statutory health insurance fund: “Services must be sufficient, appropriate, and cost-effective; they must not go beyond the indispensable minimum” [10].

The knowledge on the impact of the use of antiretroviral treatment on direct costs within the German health care system is scarce [11], [12]. A long-term follow-up of direct costs for cART regimens in the nationwide German ClinSurv multi-centre cohort will allow elucidation of the impact of more recently licensed antiretroviral drugs and the evolution of treatment guidelines for this cost-setting, economic and main part of the care of HIV/AIDS. The main aim of this study was to determine the trend dynamics of direct costs associated with the implementation of cART in clinical practice that reflect the prescribing patterns of particular antiretroviral drug regimens. The second aim was to estimate the potential impact of the introduction of generic antiretrovirals on the direct treatment costs of HIV in the German pharmaceutical market.

## Methods

By law (National protection against Infection act, IFSG, 2001) the Federal Commissioner for Data Protection and Freedom of Information recommended the clinical surveillance of HIV-infection in Germany. The Clinical Surveillance of HIV Disease, ClinSurv study, was approved by the Federal Commissioner for Data Protection and Freedom of Information. No personal data and no biomaterials are collected. The Federal Commissioner for Data Protection and Freedom of Information waived the need for written consent, because data are reported anonymously to the German Public Health Institute, the Robert Koch-Institute. The ClinSurv study is an ongoing, prospective, long-term observational cohort study. The study design is described in detail elsewhere [12]. Up to the present study, a total of 14,377 HIV-infected patients monitored at 11 clinical centres in different, predominantly urban areas in Germany have been enrolled in the study and consecutively monitored since January 1^st^ 1999.

Due to the fact that the cohort enrolment was initiated in the pre-cART era, patients on single-drug cART or double-combination cART, as well as patients on cART, were included in the cohort. A biannual data collection was performed using local electronic databases that capture information on antiretroviral therapy, treatment initiation and changes in treatment since the first visit to the reporting centres. Immunologic, virologic and demographic data, AIDS-defining diagnoses and information on cART are reported. All time-related variables were collected and referenced according to their days of occurrence. The data were validated and monitored manually as well as electronically at the coordinating centre. The source data were verified and double reporting was excluded.

The history of antiretroviral therapy administration was documented individually by beginning and end dates and the administered dosages were calculated on a daily basis for every single antiretroviral drug. The active substances in each particular pharmaceutical ingredient are categorized by their International Non-proprietary Names (INN). All INN were categorized within their appropriate substance classes, which are defined by their mode of action (Table 1). In addition, the ingredients of co-formulated drugs were assigned to the single antiretroviral agents included.

**Table 1:** Licensed antiretroviral drugs and their assigned substance classes.

Antiretroviral substance (INN)	Abbreviation	Class (defined by mode of action)	German launch date (month/year)
Abacavir	ABC	nRTI	07/1999
Didanosine	ddI	nRTI	9/2000
Emtricitabine	FTC	nRTI	10/2003
Lamivudine	3TC	nRTI	08/1996
Stavudine	d4T	nRTI	05/1996
Tenofovir	TDF	nRTI	11/2001
Zalcitabine	ddC	nRTI	Before 1996
Zidovudine	AZT	nRTI	Before 1996
Efavirenz	EFV	NNRTI	5/1999
Nevirapine	NVP	NNRTI	2/1998
Amprenavir	APV	PI	10/2000
Atazanavir	ATV	PI	03/2004
Darunavir	DRV	PI	02/2007
Fosamprenavir	FPV	PI	07/2004
Indinavir	IDV	PI	10/1996
Lopinavir	LPV	PI	03/2001
Nelfinavir	NLF	PI	01/1998
Ritonavir	RTV	PI	08/1996
Saquinavir	SQV	PI	08/1998
Tipranavir	TPV	PI	10/2005
Enfuvirtide	T20	FI	5/2003
Maraviroc	MVC	CCRI	09/2007
Raltegravir	RAL	INSTI	12/2007
Hydroxyurea	HU	RRI	Before 1996

Abbreviations see glossary.

Treatment entities are defined as substance regimen (SR) by lists of pharmaceutical ingredients or as class regimen (CR) by the list of their dedicated substance classes for all individual antiretrovirals taken at the same time. The amounts of all single drugs and the numbers of antiretrovirals representative for each single substance class were calculated and defined as the daily swallowed pill burden. The regimens were defined as PI-boosted if ritonavir was added to another PI in a daily dose of less than or equal to 400 mg per day, whereas ritonavir was regarded as an antiretroviral active drug when given in a higher dosage. In order to assign an individually appropriate treatment line, the cART history was retrospectively documented in all patients who started cART before entering the study. Any qualitative change of treatment regimen defined a subsequent regimen and via this an incrementally increasing treatment line. If it was known that a patient was treatment naive, a regimen was defined as the first line regimen. Treatment naive patients were not included in the cost analysis unless at the point of initiation. Treatment interruption was defined as any period without the intake of cART by a previously treated patient.

### Allocation of direct treatment costs

Allocation of direct costs for cART was calculated by daily standard doses for all elements of an entire regimen (treatment days) on the basis of historical and actual office sales prices of the original pharmaceuticals distributed by the license holder; the prices include German taxes as given in the German pharmaceutical online database (Lauer Taxe; www.lauer-fischer.de). No direct costs were assigned for particular antiretroviral substances during periods of early access programmes before licensing of the drug in Germany. Treatment costs were calculated exclusively for the days under treatment with at least one antiretroviral drug, including hydroxyurea, which was assumed to have antiretroviral effects in the late 1990s. The potential impact of discarded drugs when regimens were changed and when non-adherence (e.g. forgotten doses) was reported were not included in the cost analysis. The mean costs over time were adjusted for the annual German consumer price indices (German Federal Statistical Office 2010, www.destatis.de; 1996 = 100%). The German national annual increments of the consumer price index in percentages were: 1996: 1.4%, 1997: 1.9%; 1998: 1.0%; 1999: 0.6%; 2000: 1.4%; 2001: 1.9%; 2002: 1.5%; 2003: 1.0%; 2004: 1.7%; 2005: 1.5%; 2006: 1.6%; 2007: 2.3%; 2008: 2.6%.

### Calculation of cost saving strategies with NNRTI or potential generics/sensitivity analysis

Estimates of the cost impact of the use of a preferred NNRTI instead of PI, and rates of 50% and 90% substitution of PI(/r) by NNRTI in first line therapy were calculated. Assuming that in the near future generic zidovudine, lamivudine, nevirapine and efavirenz will be available in Germany, the potential cost impact of using these generic drugs was modelled. Scenarios with a price reduction between 20% and 90%, as compared to the recent prices for branded drugs, and prescription rates of 20% up to 90% for applicable generic drugs were calculated. For the purposes of this analysis it was also assumed that branded emtricitabine could be substituted by generic lamivudine in certain cases.

### Statistics

Quarterly datasets were determined for the observation period. Statistical analysis was performed using PASW 18 statistical software. Mean values and their standard deviations were calculated. Differences between the means were compared by ANOVA tests. The p-values of <0.05 were considered as denoting statistical significance, and all tests of significance were two-sided.

## Results

### Study population of the ClinSurv Cohort

In total, 10,190 HIV-positive patients observed in the German ClinSurv Cohort were enrolled in the study during the period of January 1^st^ 1996 to December 31^st^ 2008. Nearly 80% of these patients were male, 75% were German born, and over 60% were symptomatic according to CDC-B and CDC-C classification (Table 2). The women were younger (median of 32 years old) than the men (median 38 years old). The period between the first diagnosis of HIV in women and their first clinical site visit in one of the participating study centres was shorter than for the men. The women were in earlier stages of HIV disease and had histories with less advanced treatment lines than the men. Any PI-based regimen was prescribed less frequently (42% of treatment days) for the women than for the men (50%). Treatment lines, stages of HIV disease, time of first diagnosis, and age also differed between groups, with distinct transmission risks and different ethnic groups.

**Table 2:** Patients' characteristics at the time when their first cART regimen was documented within the ClinSurv cohort.

	n (%)
Total number of cases	10,190
**Gender**	
Female	2062 (20.2%)
Male	8128 (79.8%)
**Risk**	
Men who have sex with men (MSM)	5112 (50.2%)
Intravenous drug use (IDU)	973 (9.5%)
Transfusion/Haemophilia	186 (1.8%)
Heterosexual	1421 (13.9)
Origin from high prevalence region	1300 (12.8%)
Vertical transmission	20 (0.2%)
Unknown/other	1178 (11.6%)
**Origin**	
Germany	7653 (75.1%)
Western Europe	353 (3.5%)
Central- & Eastern Europe	452 (4.4%)
Sub-Saharan Africa	1025 (10.1%)
Asia/Northern Africa	326 (3.2%)
Americas/Caribbean	179 (1.8%)
Unknown/other	202 (2.0%)
**CDC-Stage**	
CDC-A/unknown	4038 (39.6%)
CDC-B	2105 (20.7%)
CDC-C	4047 (39.7%)
**Age (years)**	
Mean (+/− SD)	38 (+/−10.7%)
Median (range: min-max)	36 (2–81%)
**Year of first HIV diagnosis**	
Range (min - max)	1984–2008
**Observation period**	
Range	1.1.1996–31.12.2008

**Figure 1:**
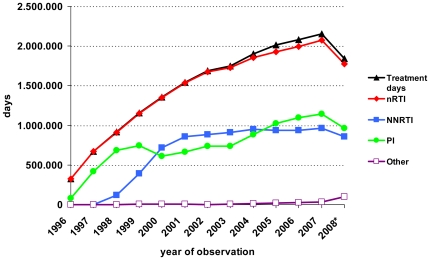
Cumulative use (in days) of antiretroviral classes by year. (* The decrease of treatment days in 2008 is explained by a reporting delay for some of the most recent cases within the database at the end of the observation period).

During the 13-year follow-up period 21,387,427 individual treatment days were covered (Figure 1). A cumulative direct cost of €812,877,356 for antiretroviral drugs was calculated. The average cost per day was €42.08 (€7.52–217.70). Out of 21.39 million documented treatment days, 1.99 million were assigned to treatment interruptions. The average direct costs for treatment days were decreased by 9.7% (to €38.00) when these interruptions were included in the analysis. Treatment interruptions appeared less frequently in 1997 (5.3%), were predominantly reported in 2003–2004 (12.1% per year), and decreased again in 2008 (6.3%).

### Composition of treatment regimens in the ClinSurv Cohort

**Figure 2:**
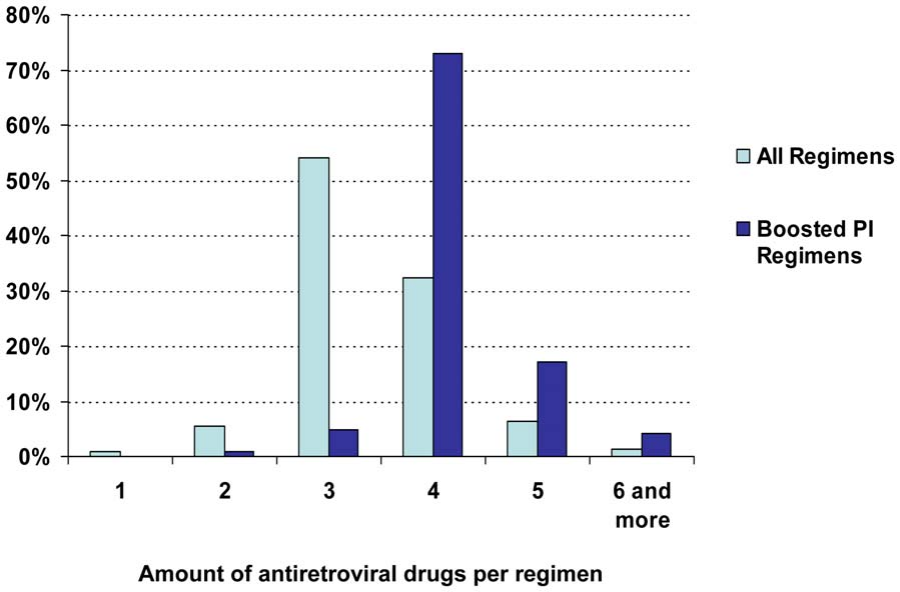
Distribution of the amount of different antiretrovirals per regimen in % of all treatment days. Observation period 1996–2008.

The mean number of drugs increased continuously during the observation period (1996: 2.05 vs. 2008: 3.62). Each regimen was comprised of 3.4 substances (mean +/−0.79), including ritonavir. Ritonavir appeared in 33% of all regimens prescribed. The regimens with boosted PI/r contained significantly more antiretroviral components (4.2+/−0.65) and ARV classes (2.07+/−0.39) than non-boosted regimens (substances: 3.05+/−0.54; ARV classes: 1.87+/−0.43, p<0.0001; Figure 2).

A predominance (51.0%) of PI/r-based treatment regimens was reported in the entire cohort (n = 249,340 treatment quarters). When analysing patients exclusively on their first line treatments (n = 62,966 treatment quarters), PI based regimens (52.3%) predominate as well NNRTI-based regimens, but NNRTI outweigh the P based regimens in second- (51.8%; n = 39,780 treatment quarters) and third-line regimens (50.6%; n = 33,333 treatment quarters). NNRTIs were available later than PI, and therefore the use of NNRTI increased over time in early treatment lines. In 1998: 12.7% in first, 14.6% in second, and 12.1% in third line treatment as compared to 51.2%, 58.9% and 54.2% in 2008. Until 2001, virtually all regimens consistently contained one or more nRTIs. In contrast, in later treatment, the lines that switched from NNRTI to PI/r prevailed.

**Figure 3:**
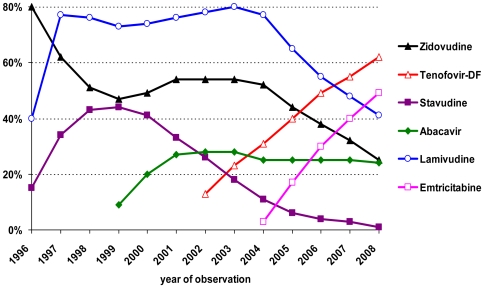
Market share of selected antiretrovirals of the nRTI-class (% of all treatment days per year).

The proportion of more recently licensed drug classes (fusion inhibitor FI, integrase strand transfer inhibitor INSTI, chemokine co-receptor inhibitor: CCRI) was cumulatively less than 5% among all of the ARV regimens reported in 2008, with a tendency to increase. Overall, 2.2 different single antiretroviral substance regimens were prescribed in the cohort and 197 different drug class regimens (Table 3) were composed. However, the top ten class regimens covered more than 90% of all prescribed regimens. The top ten drug compositions represented more than a third of all drug regimens. Zidovudine/lamivudine-containing regimens were the dominant regimens during the observation period. They decreased from 80% in 1996 to 25% in 2008, whereas tenofovir, comprising a backbone, increased from 0% to 62% (Figure 3).

**Table 3:** Top ten antiretroviral substance regimens (n = 2226) and class regimens (n = 197).

	Substance regimen	Number of regimen quarters	Cumulative frequencies in %	Class regimen	Number of regimen quarters	Cumulative frequencies in %
1	AZT/3TC/NVP	19,558	7.8	NNRTI + 2 nRTI	83,874	33.6
2	AZT/3TC/EFV	15,745	14.2	PI/r + 2 nRTI	56,417	56.3
3	TDF/FTC/EFV	10,627	18.4	PI + 2 nRTI	31,940	69.1
4	AZT/3TC/LPV/r	9665	22.3	3 nRTI	12,955	74.3
5	AZT/3TC/ABC	9006	25.9	2 nRTI	11,985	79,1
6	TDF/FTC/LPV/r	6798	28.6	NNRTI + 3 nRTI	9685	83.0
7	AZT/3TC/IDV	6090	31.1	PI/r + 3 nRTI	8307	86.3
8	AZT/3TC	5605	33.3	2 PI + 2 nRTI	5502	88.5
9	TDF/FTC/ATV/r	5094	35.4	PI/r + NNRTI + 2 nRTI	3395	89.9
10	TDF/FTC/NVP	5088	37.4	PI + NNRTI + 2 nRTI	2842	91.0

Up to 48 treatment lines were documented within the observation period. The means and maximums of treatment lines increased during the 13 year follow-up period. The proportion of patients who received a first line regimen was highest at the start of the observation period in 1996 (39.6%) and lowest in 2008 (21.6%; Table 4).

**Table 4:** Regimen lines over time (observed patient quarters in selected years).

	1996	2000	2004	2008
Number of observed patient quarters per year	5216	17306	23776	25570
% with 1^st^-line cART	39.3%	26.8%	25.7%	21.6%
Mean # of treatment line	2.5	3.9	4.7	5.0
Maximum # of treatment line	20	37	40	48

### Drug costs – status quo in the German ClinSurv Cohort

**Figure 4:**
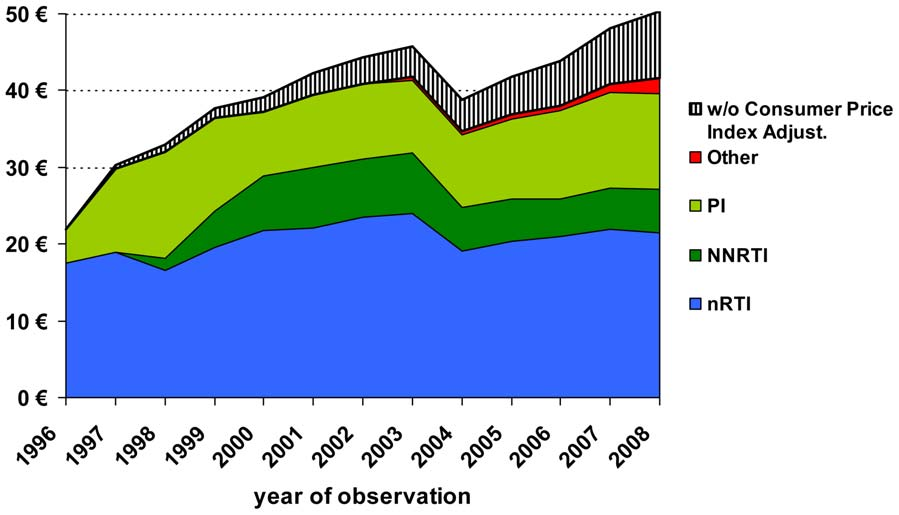
Mean daily treatment costs of antiretrovirals by class and year after adjustment for the German consumer price index (1996 = 100), annual increments of consumer price index in %: 1996: 1.4%, 1997: 1.9%; 1998: 1.0%; 1999: 0.6%; 2000: 1,4%; 2001: 1.9%; 2002: 1.5%; 2003: 1.0%; 2004: 1.7%; 2005: 1.5%; 2006: 1.6%; 2007: 2.3%; 2008: 2.6%.

Between 1996 and 1999 the mean daily drug costs for an entire regimen increased substantially (+72%: €21.89+/−9.57 to €37.70+/−9.21) by the introduction of triple c-ART. Since 1999 the increase in daily drug costs was more moderate until 2008 (33%: € 50.05+/−16.47). As a consequence of a 16% price cut enacted by law, a sharp decline of 15.4% was observed between 2003 (€45.82) and 2004 (€38.75). After adjusting costs for the annual German consumer price indices (German Federal Statistical Office 2010, 1996 = 100%), the real increase in daily drug prices for the prescribed cART regimen in the cohort was 13.5% (1999: mean: €36.39/d and 2008: mean: €41.32/d, Figure 4).

**Figure 5:**
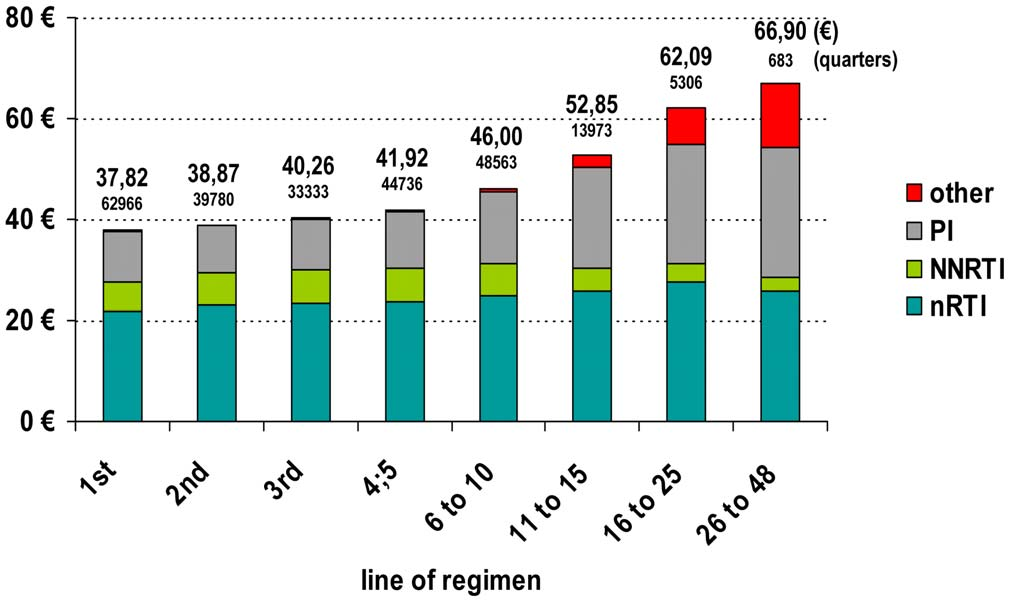
Direct costs of antiretrovirals per line of regimen (pharmacy prices including taxes in Euros. Fine-printed numbers represent sample sizes. p<0.0001 for differences between regimen line groups).

Substantial differences in average drug prices existed between different antiretroviral classes. Within the same drug classes, differences between substances were comparatively small. Patients in advanced treatment lines received more complex and more expensive regimens. Expenditures for PI, INSTI, CCRI and Fusion Inhibitors (FI) increased substantially in advanced treatment lines, but remained constant for nRTI, and decreased for NNRTI (Figure 5).

In symptomatic HIV-disease stages the mean daily drug costs for cART were higher (CDC-B: €42.33+/−14.34, n = 53,239 regimen quarters; CDC-C: €44.45+/−15.00, n = 86,615 regimen quarters) compared to patients with asymptomatic or unknown stages of HIV (€40.09+/−11.84, n = 109,486 regimen quarters; p<0.0001). Differences were less pronounced than those between treatment regimen lines, reflecting the fact that complex treatment histories were also seen in less advanced stages of the disease.

**Figure 6:**
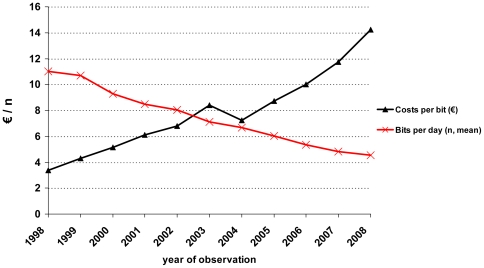
Means of daily taken pills (bit) and average costs of each single antiretroviral pill by year.

The direct costs of cART were lower for females (€40.42+/−12.65) than males (€42.46+/−13.88, p<0.0001). The amount of daily swallowed pills (pill burden) decreased via the incremental use of fixed drug combinations from 11.02 bits/day (+/−5.11) in 1998 to 4.53 bits/day (+/−2.63) in 2008. In parallel, the proportion of regimens with three or fewer pills per day increased from 5.6% to 41.1% in 2008, hence the price per bit increased substantially from €3.38 to €14.22 per day during that time (Figure 6).

### Potential cost savings by modelling the different treatment strategies: Cost impact of NNRTI vs. PI in first line cART

Overall, 4.9 million out of 19.4 million treatment days in this cohort (25.3%) referred to patients receiving first line therapy. The proportions of NNRTI- and PI-comprising first line regimens were comparable (NNRT: 41.4%; PI/r 45.7%). In contrast, the mean daily costs of the PI/r regimen were higher than the costs of NNRTI-comprising regimens (PI/r: €22.07; NNRTI: €14.32). Hypothetically, assuming that at least 50% and as much as 90% of a PI/r-comprising first line regimen could be substituted by less expensive NNRTIs, this change would result in cumulative savings of 10.9% to 19.8% respectively, of the daily treatment costs.

### Potential cost impact of generic antiretrovirals

Of all of the direct costs of antiretroviral drugs, 44.4% are related to the use of zidovudine, lamivudine, emtricitabine, nevirapine and efavirenz, which were exclusively available as branded drugs within the observation period. These drugs might be candidates for substitution by generic drugs or equivalents in the near future in Germany. The relative cost impact of these potentially replaceable branded drugs decreased from 44.1% in 2005 to 34.9% in 2008 for all of the mean direct costs of antiretrovirals (Table 5).

**Table 5:** Total costs of antiretroviral therapy by selected antiretroviral components potentially replaceable by generics in future (cumulative sum in € per period, and % of costs of the entire regimen).

	1996–2008	2005	2006	2007	2008
Observed patient quarters (n)	249,340	25,406	25,210	25,823	25,570
AZT	96,711,539 (11.9%)	9,763,763 (11.7%)	8,614,916 (9.5%)	8,166,949 (8.0%)	5,698236 (6.2%)
3TC & FTC	143,208,236 (17.6%)	14,510,090 (17.4%)	16,068,364 (17.7%)	17,694,612 (17.3%)	15,732,138 (17.1%)
NVP & EFV	120,645,732 (14.8)	12,504,141 (15.0%)	11,707,680 (12.9%)	13,850,164 (13.6%)	10,643,741 (11.6%)
AZT, 3TC, FTC, NVP, and EFV	360,565,506 (44.4%)	36,777,993 (44.1%)	36,390,960 (40.2%)	39,711,724 (38.9%)	32,074,114 (34.9%)
All other ARVs	452,311,850 (55.6%)	46,566,447 (55.9%)	54,173,304 (59.8%)	62,455,933 (61.1%)	59,740,7217 (65.1%)
Entire regimens	812,877,356 (100%)	83,344,440 (100%)	90,564,264 (100%)	102,167,657 (100%)	91,814,831 (100%)

Using different hypothetical price cuts related to the use of generic drugs (from 20% up to 90% as compared to recent prices of the branded drugs) and different rates of substitution of the branded ARVs by generics (20% to 90%), the potential impact for cost savings was calculated as ranging from 1.6% up to 31.8% of the direct costs of all prescribed antiretrovirals (modelling utilized the most recent data from 2008, Table 6).

**Table 6:** Daily costs of antiretroviral therapy components (Euros/per day).

		1996–2008	2005	2006	2007	2008
Mean daily costs in Euros of the entire regimen(+/− standard deviation)		€ 42.08 (+/−13.68)	€ 41.83 (+/−11.74)	€ 43.89 (+/−13.51)	€ 48.13 (+/−14.89)	€ 50.05 (+/−16.47)
Potential for savings by generics in % of entire direct costs, assuming:	30% price reduction	14.5%	14.3%	13.4%	12.8%	11.8%
	60% price reduction	29.0%	28.5%	26.8%	25.7%	23.6%
	90% price reduction	43.5%	42.8%	40.2%	38.5%	35.4%
Saving in % of entire direct costs, assuming:	Progressive scenario: 90% reduction, 90% substitution	**39.1%**	**38.5%**	**36.2%**	**34.7%**	**31.8%**
	Conservative scenario: 20% reduction, 20% substitution	**1.9%**	**1.9%**	**1.8%**	**1.7%**	**1.6%**

Sensitivity analysis modelling of potential price reduction in % for different scenarios of price reduction in the case of availability as a generic drug and for different quotas of patients treated with generic drugs (standard deviation for costs; mean cost saving of the entire regimen in %).

## Discussion

The German health care system has set up some unique regulations regarding pricing policies for over-the-counter drugs (or the OTC market) and for drugs only available on prescription [13]. Besides this, manufacturers have the right to fix branded drug prices at desired levels largely without negotiation with the authorities or insurance companies. Therefore, German drug prices have an important impact on the EU-wide market. In addition, branded drugs are sold in German pharmacies nationwide on the basis of the price, which is fixed by the manufacturer. Both features are reasons for the comparably higher pricing of branded drugs in Germany [8]. However, statutory discounts and reference pricing lists have recently been modified by legal regulations [14]. A statutorily ordered price cut of 16% in 2004 led to a sustainable reduction of the increase in antiretroviral drug prices for several years in the cohort studied (Figure 4).

### Use of substance classes and the reflectance of treatment guidelines

Cost analyses take the cost-bearer's view and therefore the costs of antiretrovirals given in clinical trials or expanded access programmes are disregarded. Thus, the monetary assessment of cART in this study should almost exclusively reflect the antiretroviral regimens in clinical day life, which should be geared towards current national treatment guidelines [4]. The primary goals of these guidelines are the initiation of first line antiretroviral therapy and the implementation of principles for proceeding in cases of treatment failure.

Within a clinical setting there might be a need to change an antiretroviral regimen due to several reasons, which can result in a rather short median duration of first line regimens of a couple of months [15]. This might explain the considerable diversity in antiretroviral treatment within the ClinSurv cohort, resulting in more than 2000 different antiretroviral substance regimens. However, 37.4% of these regimens were covered by the top ten SRs and 80.5% by the top one hundred, respectively. Analyses of the patterns of the prescribed cART regimens demonstrated an almost complete compliance with the actual treatment guidelines at any time.

Variations and clusters of certain regimens between participating centres were obviously determined by on-going studies or by the pre-selection of patients with particular co-morbidities or more advanced stages of HIV at some of the ClinSurv centres. For example, boosted double PI-regimens were not recommended by German treatment guidelines but they still accounted for a 1.0% of all documented regimens within the cohort. These patients predominantly presented at particular study centres.

Treatment recommendations from the treatment guidelines reflected rather data regarding efficacy and tolerability from clinical trials than health and economic considerations [16]. Yet, in Germany, physicians are obliged to prescribe the economically most advantageous alternative within the publicly funded health care system. However, this stipulation is restricted to equipollent treatment options, which are hardly defined in clinical practice. On the other hand, until recently, treatment guidelines did not discuss the cost impacts of the given recommendations. The NNRTI- and PI/r-based drug regimens are equally recommended for first line treatment in recent guidelines [4], [5]. The mean daily costs of PI/r were found to be 53.8% greater than for NNRTI within the treatment days in this cohort, which referred to patients who received first line therapy. Among the initial regimens, the distribution of NNRTI and PI/r-based regimens was almost balanced.

Regarding the statutory German efficiency principle for drug prescription it is noteworthy that in the cohort study the proportion of NNRTI-based cART increased rapidly at the end of the 1990s (Figure 1), resulting in a preponderance since 2000 for the first and second line regimen over the more expensive PI/r-based cART. Interestingly, PI/r use was more common in the first than in the second line regimen. A potential rationale for preferring a PI/r-based induction therapy is the improved resistance of the PI class in cases with a high viral load at the start of treatment [17]. Accordingly, when analysing the changes following the administration of first line therapy, we found fewer changes from NNRTI to PI/r-based regimens than PI/r-induced patients switching to a second line NNRTI regimen, soon after their HIV viral loads were beyond the limit of detection. Assuming that, hypothetically, up to 90% of PI/rs could be substituted by less expensive NNRTIs, this would result in cumulative savings of up to 20% of the daily treatment costs in the group of patients on the first line treatment. The potential impact of such an approach on the long-term outcome and cost efficacy needs further investigation. Additionally, a putatively more convenient antiretroviral regimen or a specific desire of the patient could result in the prescription of more expensive regimens. All these reasons might additionally explain the considerable heterogeneity in the documented regimens within this study.

Nucleoside/nucleotide analogues were used as the backbone of virtually all regimens during the whole observation period. However, the proportion of nRTI-sparing regimens increased with the availability of new drug classes from 2003 and remained at more than 1% until 2008 (Figure 1). Within the nRTI class, zidovudine and lamivudine were predominantly used in the beginning of the observation period. Following the release of tenofovir and emtricitabine in 2001 and 2003, respectively, zidovudine-containing backbones decreased and tenofovir-based backbones radically increased thereafter (Figure 3). The nRTIs are cheap compared to most other antiretroviral drug classes. Nevertheless, the cost impact of nRTIs is high (Figure 4) because of the widespread use of double-nRTI-comprising regimens.

From 2003, newly licensed antiretroviral drug classes (FI, INSTI, CCRI) became available. They were primarily licensed for use in treatment-experienced patients exclusively. Cumulatively, these new drugs accounted for less than 5% of ARV-regimens in 2008, but with an increasing tendency (Figure 2). Virtually all nRTI-sparing regimens consisted of one ore more than one PI/r and more recently they increasingly consisted of agents from the new drug classes. Using the opportunity provided by the increasing number of options, more regimens avoided the use of nRTI drugs when the new drug classes became available. Prices for drugs from the new antiretroviral drug classes and boosted PI are relatively high compared to nRTIs or NNRTIs; therefore, the use of nRTI-sparing treatment regimens remains a relatively expensive alternative.

### Cost impact of cART

Including the effects of the statutory 16% price cut for pharmaceuticals in Germany in 2004 and adjusting for the ascertained annual German consumer price indices (German Federal Statistical Office 2010, www.destatis.de; 1996 = 100%), the real increase in daily drug prices for the prescribed cART regimens in the cohort was 13.5% within the studied 10-year period (Figure 4). This increase is comparably moderate when considering that the many recently licensed antiretroviral drugs are significantly more expensive than those licensed ten or fifteen years ago. Substantial differences in drug prices exist between different antiretroviral classes but the increase over time within distinct antiretroviral classes was moderate, indicating that market prices of the subsequently licensed antiretrovirals seem to be orientated towards their competitors within rather than beyond the limits of their classes.

### Mapping of treatment costs for HIV in the German health care system

Since 2009, all public health insurers in Germany have to transfer the received insurance contributions to a governmental stock. Out of this stock, they prospectively retrieve a capitation of approximately €2000 and particular lump sums within a risk structure compensation scheme (Morbi-RSA). In 2010, the insurers retrieved a Morbi-RSA of €18,455.18 per year and capita for each HIV-infected patient receiving antiretroviral therapy. This study found that the HIV-specific Morbi-RSA nearly exactly covered the calculated mean costs of ARV regimens (€18,268/year) in 2008. However, drug prices have risen since 2008 and a lump sum, which exclusively covers the direct costs of cART, does not cover the direct costs of concomitant drug therapy, hospitalization or regular check-ups and therefore might indicate an underfunding for people living with HIV and AIDS (PLWHA) by the Morbi-RSA. This could cause discrimination, especially of PLWHA in the advanced stages of HIV within the German health care system in the future, especially if the Morbi-RSA is not adapted adequately. Analysis of data from German health insurance companies potentially underestimates the cost impact of cART in PLWHA. Insurance databases include a significant proportion of single prescriptions of antiretroviral drugs, presumably for post-exposure prophylaxis [18]. This underlines the impact of treatment cohort data for the calculation of lump sums such as the Morbi-RSA in the future.

### Justification of the use of generic antiretrovirals instead of branded drugs

Patent protection allows the license holder exclusive marketing and price negotiations for a defined time period after the approval of a new drug and this is regulated by national and international laws. Product licences for the treatment of HIV/AIDS, cancer and neurodegenerative diseases will have a longer period of exclusivity compared to other drugs due to different approval systems in the EU (10 years vs. 8 years; www.emea.com). After that third party suppliers are allowed to claim a license for a chemically identical drug.. All generic drugs approved by the EMEA and FDA have the same high quality of ingredients, strength, purity and stability as brand-name drugs. Generic drug manufacturers must show that a generic drug is bioequivalent to the brand-name drug, which means the generic version delivers the same amount of active ingredients into a patient's bloodstream in the same amount of time as the brand-name drug. They must have the same dosage form (for example tablets, liquids) and must be administered in the same way. Generic drug labelling must be essentially the same and generic drug manufacturers must fully document the generic drug's chemistry, manufacturing steps and quality control measures. Moreover, the generic manufacturing, packaging, and testing sites must pass the same quality standards as those of brand name drugs (FDA Requirements for Generic drugs; http://www.fda.gov/Drugs/ResourcesForYou/Consumers/ucm143545.htm#require). As for all medicines, the safety of generic medicines continues to be monitored after authorisation. Each company is required to set up systems to monitor the safety of all medicines that it markets. Regulatory authorities may also perform an inspection of these monitoring systems. If specific safety precautions have to be considered when taking the reference medicine, the same precautions will generally also be required for the generic medicine (questions and answers on generic medicines; EMEA 17 March 2011 EMA/393905/2006 Rev. 1).

However, considerable differences in the efficacy and tolerability of generic drugs had been found [19], [20] despite of pharmacokinetic bioequivalence. Based on this, additional studies should be demanded by regulatory authorities assuring the pharmacodynamic bioequivalence of generic drugs by additional clinical studies.

Therefore an exchange of branded drugs by generics without a proven bioequivalence might be problematic at least in individual cases. In the context of cART the adherence to treatment is crucial for the long term success [21] Hence it might be harmful to change fixed antiretroviral combination drug therapies to the (generic) single components. Considerable concerns may arise in case of exchanging similar but not identical drugs motivated only by the potential of cost savings, as discussed for the antiretroviral drugs lamivudine and emtricitabine, which had been included as one additional change option in the analysis. However, even the exchange of different but similar effective drugs within the same indication had been demanded as a consequence of the *economic efficiency principle* within the German Social Insurance Code, although the harm and benefit profiles of target and alternative drugs within such an approach remains controversial [22]. Therefore from a scientific point of view determining the impact on long-term outcome of drug exchange is a rational approach before its widespread use for economic reasons should be claimed. To avoid unwanted health system and health effects policy makers should assure or fund adequate studies in this field.

### Cost impact scenarios for the potential introduction of generic antiretrovirals

Until recently, all antiretroviral drugs in Germany were protected by patents and were exclusively available as branded labels. However, patent protection for zidovudine (AZT) and lamivudine (3TC) has recently expired and it will expire for nevirapine (NVP) and efavirenz (EFV) in the near future. In 2010, 3TC became available as a generic drug within the European Union (EU) exclusively. In Spain, price reductions of up to 55% can be found compared to branded 3TC (PMFarma 2010; www.pmfarma.es).

Generics are able to induce substantial price reductions for pharmaceuticals. The impact of antiretroviral generics on the direct costs of cART depends on the willingness to prescribe them broadly [8], [23].

Assuming that generic drugs will become widely available and will have an equivalent bioavailability and tolerability as branded drugs, a high penetration of the market segment will possibly be achieved. In addition with the availability of generic lamivudine, an economically based substitution of emtricitabine by generic lamivudine might also occur. The cytidine analogues emtricitabine and lamivudine have a similar resistance profile [24] and both are generally well tolerated [25]. However, different in vitro and in vivo activities and pharmacokinetic properties indicate that these two drugs might not be mutually exchangeable. [26]. Of all drug costs in the years 2005 to 2008, 35% to 44% came from drugs suitable for exchanging with generics (Table 6). With an exchange rate of 20% to 90% and price cuts from 20% to 90%, potential cost savings of 1.6% to 31.8% would result within these presumed ranges. This potentially equals mean annual savings of €292.29 up to €6,466.96 per patient. It was estimated that, in Germany, approximately 35,000 people received cART in 2008 [27]. Cost savings for the German health care system based on the introduction of generic antiretroviral drugs will be high (10 million Euros to 200 million Euros per year). Moreover, indirect cost savings might also result in the reduction of the average price of the brand name drugs that are still purchased [23]. Consequently, the introduction of generic antiretroviral drugs would potentially change the recent market situation substantially.

However, some disadvantages may arise by the introduction of generic drugs. There might be an increase in the pill burden caused by the unbundling of branded co-formulations. This might influence the adherence of patients. Although there is evidence that a high pill burden, a high frequency of dosing or both inversely correlate with patient adherence, there is little evidence for a certain threshold in the number of items swallowed daily or an advantage of the administration frequency as long as it is below three times per day. Therefore, it remains more crucial to find an individually tolerable drug combination than to minimize the pill burden [28]. But adherence is highly correlated with the long-term success of antiretroviral therapy. Therefore, efforts are needed to ensure that a change between branded and generic antiretroviral drugs will not adversely affect individual adherence. As a matter of course in cases with a known intolerability to elements of an alternative drug, an exchange is a priori contraindicated.

Assuming that the number of PLWHA in Germany will increase and as recent guidelines recommend treatment at earlier stages of asymptomatic HIV infection and at CD4+ T cell counts of less than 500/µl, many more patients will receive cART in the future. The German nationwide potential for annual cost savings by choosing economically priced antiretroviral regimens could increase up to more than 500 million euros per annum within the next few years. But this goal will be hard to achieve. Even if patients and physicians could be incentivized to consider economically alternative regimens, they will often choose a more expensive alternative [29]. Beneficiaries of less expensive treatment regimens would be the foremost payers. Hence, stakeholders have to consider how to redirect at least part of the savings to finance incremental costs for medical progress in the future.

Choosing economical alternatives in antiretroviral treatment does not necessarily mean that the pharmaceutical industry will lose earnings and market share in the future.

The development of new innovative antiretroviral co-formulations will be simplified by the availability of generic antiretroviral drugs.. This could result in advantageous treatment options for the patients. By this way, license holders of still branded antiretroviral drugs would get the opportunity to improve their portfolio and to increase their sales volume and profits.

Cost calculations from a representative, disease-specific clinical cohort, like ClinSurv, could have an impact on future cost savings. This approach would allow future definition of the cost impacts of particular subgroup treatments, for example patients in progressed stages of treatment, more precisely for the health care system and its stakeholders. In addition, it would allow the cost impacts of evolutionary changes in treatment guidelines and transitions within the German health care system to be evaluated in a prospective manner [30]. Therefore, accompanying ecological research using data of the ClinSurv cohort might improve the allocation of economic resources in health services and define specific goals for sustainable reforms of the health care system.

### Potential applicability of the findings in the study to other health care settings

The market for antretroviral drugs in the world is subdivided into at least three parts: By international policies, funding, gradual national prizing, and waiver of license fees by some patent holding pharmaceutical companies in middle- and low-income countries the drug prices vary considerably from those in the high-income countries. As discussed above the data in this study are generated from the German ClinSurv cohort using the nationwide official German pharmacy prices, which are by several reasons in an upper range even compared to other countries with a high per capita gross national product. Therefore data and conclusions from this study can not be generalised to the situation in low- and middle-income countries. Due to several unique characteristics of the German health care system and particular regulatory issues any comparison with other contrastable rich countries should be used cautiously.

### Limitations of the study

- The study focuses exclusively on direct cost for antiretroviral treatment. Other direct costs of treatment, indirect costs and intangible costs remain disregarded.
- Updates of treatment guidelines may be of high impact on treatment costs and may reduce the number of potentially cost-saving alternative treatment choices. Such potential effects for the future had not been calculated in the study.
- The used cohort data may reflect predominantly a real clinical life scenario but do not allow to investigate strategic issues, which would be addressed more accurately in a controlled clinical trial. Basically, there is a need for such trials, especially in the field of health economics. On the other hand policy makers and stakeholders have to deal with existing regulatory issues within the public health system. Beside the description of the recent cost impact of cART the presented study intends to describe a potential short-term economic impact on direct costs, by using generic antiretrovirals in a variable extent Remaining concerns regarding ethical issues to interchange branded antiretroviral drugs with generic antiretroviral drugs can neither be confuted nor confirmed by the study.
- Drug prices and regulatory issues are specifically taken from the German health care system and a comparison or generalisation of data and conclusions from this study to other health care settings should be performed with caution.
